# The genus *Unixenus* Jones, 1944 (Diplopoda, Penicillata, Polyxenida) in Australia

**DOI:** 10.3897/zookeys.156.2168

**Published:** 2011-12-20

**Authors:** Megan Short, Cuong Huynh

**Affiliations:** 1 Deakin University, 221 Burwood Hwy, Burwood, Melbourne, Australia

**Keywords:** Diplopoda, Penicillata, Polyxenidae, new species, Australia, distribution, taxonomy

## Abstract

The penicillate genus *Unixenus* Jones, 1944 is widespread, with species found in Africa, Madagascar, India and Australia. Each of the two Australian species was originally described from single samples from Western Australia. In this study, collections of Penicillata from museums in all states of Australia were examined to provide further details of the two described species, to revise the diagnoses for both the genus and the species, and to better understand the distribution of the two species in Australia. In addition, two new species *Unixenus karajinensis* **sp. n.** and *Unixenus corticolus* **sp. n.** are described.

## Introduction

Jones (1937) created the genus *Monoxenus* for his type species *Monoxenus padmanabhii* collected from the Trivandrum region in Southern India, later renaming it *Unixenus* Jones, 1944, because *Monoxenus* had been preoccupied by a genus in Cerambycidae (Coleoptera). Jones distinguished *Unixenus* from the genus *Monographis* Attems, 1907 on the grounds that the new genus had shorter tergal setae and caudal trichomes with one to five hooks rather than two hooks. However, these characters can vary within a genus, for example *Propolyxenus* Silvestri, 1948 (Short and Huynh 2010). Silvestri (1948) separated *Monographis* from *Unixenus* on the basis of the absence of the tarsal spine and this absence was then used (Nguyen Duy-Jacquemin and Condé 1967) to create the two Australian species *Unixenus attemsi* Nguyen Duy-Jacquemin and Condé, 1967, based on specimens originally identified by Attems (1911) as the African *Monographis schultzei* Attems, 1909, and *Unixenus mjoebergi* (Verhoeff, 1924)originally placed in *Monographis*. Nguyen Duy-Jacquemin and Condé (1967) also reassigned two species formerly placed in the genus *Saroxenus* Cook, 1896 to the genus *Unixenus*, namely *Saroxenus broelemanni* Condé & Jacquemin, 1962 from Madagascar and *Saroxenus vuillaumei* Condé & Terver, 1963 from the Ivory Coast. In this paper, additional characteristics of the two species *Unixenus mjoebergi* and *Unixenus attemsi* are described based on examination of numerous museum specimens and fresh material collected by the authors. Two new species from Australia are also described.

## Materials and methods

Some specimens for this study were obtained by sieving samples of bark and decomposing leaf litter into a white tray and hand-picking into 70% ethanol. The majority of specimens in this study, however, came from Australian museum collections. Specimens were examined using light and scanning electron microscopy. For light microscopy, specimens were cleared in 15% potassium hydroxide, heated in a water-bath for 2 minutes at 80°C, neutralised in 20% acetic acid for 2 minutes, rinsed in distilled water and dehydrated in a series of ethanol baths prior to staining with 1% Fast Green solution to increase contrast. The head and body were separated, the body cut open with a single latero-longitudinal incision and contents removed. After rinsing in 100% ethanol, stained specimens were transferred to 100% isopropanol, then to xylene and mounted on slides with DPX synthetic resin. Scanning electron micrographs were obtained for adults of *Unixenus corticolus* sp. n., and adults and one subadult stadium VII of *Unixenus karajinensis* sp. n. The specimens were preserved in 70% ethanol prior to being gently mounted on stubs using adhesive tabs, then air-dried, sputter-coated with gold and examined with a Philips XL20 scanning electron microscope.

Specimen lengths were measured from head to telson with caudal bundle of trichomes excluded. Adults were sexed when possible. Measurements are an indication only of size as length varies with state of activity in life and state of preservation in death. Naming of the leg segments follows Manton (1956). Unless otherwise indicated, all millipedes referred to are adults (stadium VIII). Stadium VII specimens are referred to as subadult, and “immature” refers to any non-adult stadium. The trichomes in a transverse row on the telson dorsal to the caudal bundle are referred to as ornamental trichomes.

Abbreviations: AM = Australian Museum, Sydney, New South Wales; ANIC = Australian National Insect Collection, Canberra, Australian Capital Territory; MV = Museum Victoria, Melbourne, Victoria; NSW = New South Wales; Qld = Queensland; QM = Queensland Museum, Brisbane, Qld; SA = South Australia; Tas = Tasmania; Vic = Victoria; WA = Western Australia; WAM = Western Australian Museum; L = left; R = right.

## Results

### Subclass Penicillata Latreille, 1831

**Order Polyxenida Verhoeff, 1934**

**Superfamily Polyxenoidea Lucas, 1840**

**Family Polyxenidae Lucas, 1840**

**Genus *Unixenus* Jones, 1944**

#### Unixenus

Jones, 1944

http://species-id.net/wiki/Unixenus

Monoxenus  Jones, 1937: 138; Silvestri 1948: 216.
Unixenus  Jones, 1944: 94; Nguyen Duy-Jacquemin and Condé, 1967: 68.

##### Diagnosis.

The genus is typical of the family Polyxenidae with 10 tergites plus telson, 13 pairs of legs and 8 ocelli in each eye. Antennal article VII has 2 thick basiconic sensilla with 1 setiform sensillum between, and 1 coeloconic sensillum posteriorly. Gnathochilarium has long lateral palps at least 1.5 X diameter of medial palp, 20–22 simple sensilla on medial palp and 13 on lateral palp. Spine on tarsus 2 absent, replaced with small setiform sensillum. 2 or more rows of barbate trichomes posteriorly on each tergite with trichomes arranged in 2 broad clusters either side of the midline, with a small gap between clusters. Posterior row of trichomes continuous or with medial gap, anterior row often uneven, intermediate trichomes rarely in defined rows. Telson with single caudal bundle containing trichomes with 1–11 hooks, ornamental trichomes with distinct dark long trichomes *c* either side midline.

##### Type species.

*Unixenus padmanabhii* (Jones, 1937).

#### Unixenus attemsi

Nguyen Duy-Jacquemin & Condé, 1967

http://species-id.net/wiki/Unixenus_attemsi

Figs 1A 2 3C, 3D

Unixenus attemsi  Nguyen Duy-Jacquemin and Condé, 1967: 68, figs 9, 10.

##### Material examined.

Slide preparations were made of adults from the following localities: Marangaroo Conservation Area in Perth, WA, 31°49’48"S, 115°50’12"E, 9 February 2006, M. Short and C. Huynh, in *Eucalyptus* bark; Scott Creek, SA, 35°04'37"S, 138°42'29"E, 15 February 2005, M. Short and C. Huynh, litter under *Eucalyptus*; Robinvale, Vic, 34°35'S, 142°46'E, 28 October –3 November 1968, T. Weir, J. Lawrence and E. Hansen, litter under *Eucalyptus camaldulensis*, ANIC berlesate 1085. Collections made by the authors will be deposited in WAM and MV.

##### Diagnosis.

This species can be distinguished from other species in the genus by the presence of 2 transverse rows only of short barbate trichomes on collum and tergites, 3 basiconic sensilla on antennal article VI, long lateral palps on gnathochilarium (2.5 X diameter of medial palp), 1 seta on femur and no setae on tibia, funicle of leg setae with no projecting spines, telotarsus with 4–6 processes on claw, thin anterior spinous projection same length as claw, 3 ornamental trichomes *c* each side, caudal hooked trichomes with 3–11 hooks with double pointed barbs on stem of trichomes with 4 or more hooks.

**Figure 1.**
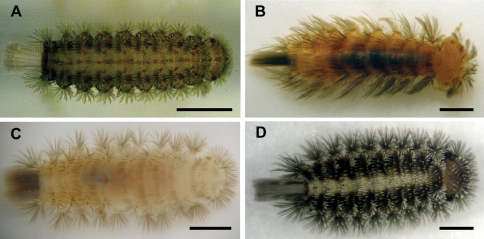
**A** *Unixenus attemsi* Nguyen Duy-Jacquemin & Condé, 1967, Scott Creek, live adult **B** *Unixenus mjoebergi* (Verhoeff, 1924), Barrow Island, WAM T71082 **C** *Unixenus karajinensis* sp. n., Wittenoom, WAM T71106 **D** *Unixenus corticolus*, sp. n., Tidal River, live adult. Scale bars = 0.5 mm.

**Figure 2.**
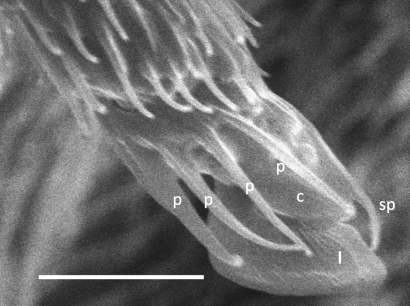
*Unixenus attemsi* Nguyen Duy-Jacquemin & Condé, 1967, adult male, Perth. Telotarsus showing 4 processes attached to the base of the claw. p = process, c = claw, sp = anterior spinous projection, l = lamella process. Scale bar = 5 µm.

**Figure 3.**
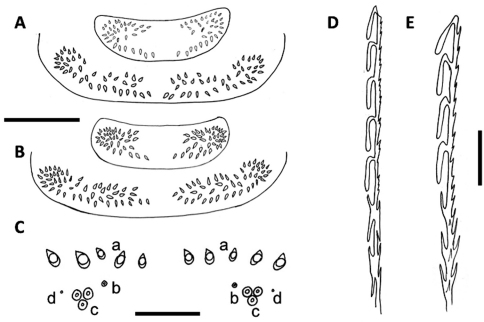
**A** Pattern of trichome insertions in collum and tergite 2 of *Unixenus mjoebergi* (Verhoeff, 1924) from Eil Eil Spring, WAM T71082 **B** Pattern of trichome insertions on collum and tergite 2 of *Unixenus mjoebergi* from Hann Tableland, QM S17167 **C** Pattern of ornamental trichome insertions *a, b, c* and circular indentations *d*, *Unixenus attemsi* Nguyen Duy-Jacquemin & Condé, 1967, adult female, Robinvale, ANIC berlesate 1085 **D** Hooked trichome with double barbs on stem, *Unixenus attemsi*, adult female, Robinvale, ANIC berlesate 1085**E**Hooked trichome with single barbs on stem, *Unixenus mjoebergi*, Barrow Island, WAMT71112. Scale bars: A & B (shared) = 200 µm; C = 30 µm, D & E = 40 µm.

##### Remarks.

The original description by Attems (1911) was very brief. A redescription of the species by Nguyen Duy-Jacquemin and Condé (1967) was based on examination of two female specimens from Torbay in southern Western Australia (the type locality) held in the Zoology Museum, Hamburg, Germany (coll. by Hamburg S.W. Australia Expedition 1905). This description is detailed and clearly illustrated, but in light of recent collection of the species from eastern Australia and examination of large numbers of the species from a range of locations in WA, the species description can now be expanded and variation in some characters recorded.

##### Additional description.

Body unpigmented with exception of darkly pigmented medial longitudinal band on dorsal surface and darkly pigmented lateral projections. Trichomes including caudal bundle colourless (Fig. 1A). Body length from Robinvale and Scott Creek specimens 1.2–1.7 mm (n = 6), Perth 1.4–1.8 mm (n = 5), Walpole-Nornalup National Park, WA, WAM T71144, 2.3 mm; caudal bundle 0.3–0.4 mm. Variation noted in number of trichomes in rows on posterior vertex of head, between specimens and between left and right (as determined by examination of trichome insertion points). Specimens examined varied with numbers each side: Perth 16–19 (n = 5), Scott Creek 12–13 (n = 4), Robinvale 9–14 (n = 7), no difference between males and females, individual maximum difference between left and right sides of vertex = 2. Clypeo-labrum as previously described, with 8 setae most commonly observed along posterior margin, in contrast to 9 described by Nguyen Duy-Jacquemin and Condé (1967).

Collum showsvariation in trichome number: Perth 19–25 each side (n = 4), Scott Creek 16–19 (n = 2), Robinvale 15–16 (n = 1). Maximum variation between right and left sides on a single individual = 2. On tergite 2, trichome numbers varied (each side of midline): Perth 23–32 (n = 4), Scott Creek 19–23 (n = 4), Robinvale 20 (n = 1). Males with 2 pairs of coxal glands, leg pairs 8 and 9. Variation observed in number of ornamental trichomes *a*: Perth 7–10*a* (each side) (n = 5), Scott Creek 6–7*a* (n = 5), Robinvale males 4–5*a* (n = 2), females 6–9*a* (n = 2). Adult females described by Nguyen Duy-Jacquemin and Condé (1967) had 10 and 12 trichomes *a* each side. Circular indentation (labelled *d*) observed adjacent and external to each cluster trichomes *c* (Fig. 3C). This structure, also illustrated in Figure 3G in Condé and Jacquemin (1962) labelled ‘*x*’, is present in all adult specimens of the genus examined but function unknown. Telotarsus with 4 processes on claw rather than 6 processes previously observed (Nguyen Duy-Jacquemin and Condé 1967) (Fig. 2).

Single caudal bundle of hooked trichomes, 2–11 hooks with barbed stems, size of hook and nature of barbed stem dependent on number of hooks: trichomes with 2–4 hooks, hooks large, all barbs on stem distal-facing similar to those in *Unixenus mjoebergi* (Fig. 3E), trichomes with 4–11 hooks, hooks smaller and with double barbs along stem proximally, with both proximal- and distal-facing points (Fig. 3D). Specimens examined by Nguyen Duy-Jacquemin and Condé (1967) had 5–7 hooks, with no description given of barbs along stem.

##### Distribution.

This species is widespread in Australia (Fig. 8) but appears to be most common in a range of habitats in southern WA. The most northerly collection is Bush Bay, WA, 25°07’03"S, 113°48’22"E, where it was collected together with *Unixenus mjoebergi* (WAM T71125). In eastern Australia the species appears to be restricted to dry woodlands. It is found under bark of *Eucalyptus* in small aggregations, often with many exuviae, as well as in dry leaf litter and under stones in treed areas on well-drained sandy or sandy loam soil.

##### Remarks.

The two Torbay specimens described by Nguyen Duy-Jacquemin and Condé (1967) are longer than almost all measured in this study, at 2.25 and 2.65 mm. Torbay is on the south coast of WA (Fig. 8) and a single specimen in the WAM collection (T71144) from Walpole-Nornalup National Park on the south coast of WA is also large (2.3 mm). All adults from eastern Australia are smaller. Smaller adults have fewer trichomes on head and vertex.

#### Unixenus mjoebergi

(Verhoeff, 1924)

http://species-id.net/wiki/Unixenus_mjoebergi

Figs 1B 3A, 3B, 3E

Monographis mjoebergi  Verhoeff, 1924: 38.
Unixenus mjoebergi  Nguyen Duy-Jacquemin and Condé, 1967: 73, figs 11, 12.

##### Material examined.

Slide preparations were made of adults from the following collections: Barrow Island, WA, 20°47'38"S, 115°27'24"E, sites 17 (2 suction samples) and 105 (2 samples: suction and Winkler), 24 April 2005, K. Edward and S. Callan, WAMT71111–4; Eil Eil Spring, WA, 19°47'S, 121°26'E, 20 September 2002, K. Edward, in litter, WAM T71082; Hann Tableland, Qld, 16°49'S, 145°11'E, 950 m, 12 December 1995, G. Monteith, pyrethrum spray on bark, open forest, QM S38765; islands in Capricornia Cays National Park, Qld, pitfall trapping by QM and Queensland Parks and Wildlife Service: Masthead Island site 1, 23°32'24"S, 151°43'44"E, 0–5 m, 5–7 October 2008, grassed areas on beach, QM S17167; Erskine Island site 2, 23°30'07"S, 151°46'23"E, 2 m, 6–8 October 2008, open grassland, QM S17561; Lady Elliot Island site 7, 24°06'50"S, 152°42'58"E, 0–5 m, 29–31 March 2008, beach bean vine thicket, QM S16031; West Hoskyn Island site 1, 23°48'32"S, 152°17'20"E, 0–5 m, 13–15 May 2008, *Casuarina* stand, QM S17052; One Tree Island site 1, 23°30'25"S, 152°05'31"E, 0–5 m, 23–25 September 2008, *Casuarina* stand, QM S17144; North West Island site 8, 23°17'31"S, 151°42'29"E, 0–5 m, 9–11 October 2008, *Casuarina* stand, QM S17609; and Eungella, 21°12'S, 148°26'E, 600 m, 18 November 1981, Gillison, ANIC berlesate 984.

##### Diagnosis.

This species can be distinguished from other species in the genus by the presence of 3 or more transverse rows of barbate trichomes on collum and tergites 2 and 3, with 2 or more rows in remaining tergites, 3 basiconic sensilla on antennal article VI, long lateral palps on gnathochilarium (2.5 X diameter of medial palp). 2 setae on femur and 1 seta on tibia, funicle of setae of coxa, prefemur and femur ridged, ridges extending as projections surrounding flagellum, telotarsus with 2 processes (anterior and posterior) on claw, anterior spinous projection longer than claw, 3 ornamental trichomes *c* each side, caudal hooked trichomes with 1–4 hooks, absence of double pointed barbs on stem.

##### Remarks.

The original description by Verhoeff (1924) was very brief and no illustrations were given. A redescription of this species by Nguyen Duy-Jacquemin and Condé (1967) was based on a single paratype adult female preserved in ethanol from the Museum of Comparative Zoology, Harvard University. This description is detailed and clearly illustrated, but in light of recent collection of the species from eastern Australia and examination of large numbers of the museum specimens from a range of locations in Western Australia and Queensland, the species distribution can now be extended and variation in some characters recorded.

##### Additional description.

No freshly collected specimens available. All specimens had been preserved in 70% ethanol for at least 18 months prior to examination, with most in ethanol for decades. Body yellow brown in colour with trichomes including caudal bundle pigmented dark brown. Average length adult (mm): Barrow Island 2.1–2.5 (n = 3), Hann Tableland 3.0–3.4 (n = 5), Capricornia Cays 2.0–2.8 (n = 15); caudal bundle 0.4–0.5 mm. No differences observed between sexes. Variation in both pattern and number of trichomes on posterior vertex of head in specimens from different populations and variation within a population between specimens, and between left and right on a single individual. Pattern of 3 oblique rows each side separated medially by broad gap as described for paratype by Nguyen Duy-Jacquemin and Condé (1967) most common, but occasionally 2 rows, and 1 specimen with 5 rows each side. Trichomes barbate and longer than those of *Unixenus attemsi*. Variation in trichome number each side as follows: Barrow Island females 22–26 (n = 2), males 20–22 (n = 2); Eil Eil Spring subadult male 18–19; Eungella subadult male 19–21; Hann Tableland subadult males 19–25 (less in posterior row) (n = 3), adult females 23–32 (n = 5); Capricornia Cays, both males and females 17–23 (n = 12), plus 1 male with 5 rows, 34 each side. Clypeo-labrum as described for paratype by Nguyen Duy-Jacquemin and Condé (1967) for all specimens examined, with 12 setae along posterior margin, except for those from Queensland mainland sites Hann Tableland and Eungella with 10 setae (n = 11); further variation was also noted with a few specimens having 3 rather than 2 lamellar teeth each side along anterior margin. Occasional variation noted in number of sensilla on gnathochilarium with 20–22 sensilla each medial round palp and 12–13 each lateral palp. The majority have 22 sensilla on medial palp in contrast to the 21 in the single specimen described by Nguyen Duy-Jacquemin and Condé (1967).

Collum similar to description in Nguyen Duy-Jacquemin and Condé (1967) with trichomes in broad lateral clusters each side of wide medial gap equal to width of cluster, a posterior row extending from lateral edge of each cluster towards the midline. Barrow Island adult specimens similar to paratype (Nguyen Duy-Jacquemin and Condé 1967) with continuous posterior row of trichomes and trichome insertion at midline. However in all other specimens examined, a median gap was present in the posterior row (Figs 3A and B). Number of trichomes each side median gap varied: Barrow Island 39–49 (n = 3), Eil Eil Spring 32–34 (n = 1, subadult male), Eungella 30–31 (n = 1, subadult male), Hann Tablelands 37–43 (n = 4), Capricornia Cays 35–57 (n = 12). In remaining tergites, only Barrow Island specimens were similar to the description by Nguyen Duy-Jacquemin and Condé (1967) with a continuous posterior row in all but tergite 10. In all other specimens examined, a median gap was present in the posterior row of most if not all tergites. Number of trichomes each side on tergite 2 variable: Barrow Island 46–60 (n = 3); Eil Eil Spring 39 (n = 1, subadult male); Eungella 36 (n = 1, subadult male); Hann Tablelands 48–66 (n = 4); Capricornia Cays 43–70 (n = 12). Tergal trichomes barbate and twice as long as those of *Unixenus attemsi*, trichomes longer in more posterior tergites. Males with coxal glands present on leg pairs 8 and 9.

Variation observed in number of ornamental trichomes *a* as follows (number given per side): Barrow Island 4–6 (n = 4, no differences between sexes); Eil Eil Spring 6 (n = 1, subadult male); Hann Tablelands: adult females 3–8 (n = 5), subadult males 8–12 (n = 3); Eungella 7–8 (n = 1, subadult male); Capricornia Cays 4–9 (n = 13, no differences between sexes). Hooked caudal trichomes with 1–4 hooks on barbed stems, barbs all distal-facing (Fig. 3E).

##### Distribution.

*Unixenus mjoebergi* has been identified in north western and north eastern Australia, including islands off the coast on either side of the continent (Fig. 8). The lack of records for central Australia and Northern Territory is possibly more a reflection of very limited and untargeted collecting effort rather than actual absence of the species from these regions. The species is found in litter of treed habitats (both open and closed forest types), and occasionally in sandy beach and sand dune habitats.

##### Remarks.

The specimens examined showed variation in a number of characters, with those of the single female adult paratype described by Nguyen Duy-Jacquemin and Condé (1967) falling within the range for each. Of particular interest is the variation in pattern of tergal trichomes with only the Barrow Island specimens showing the same pattern as the paratype. All specimens from Queensland, as well as the single specimen from Eil Eil Spring, showed a distinct gap in all or almost all tergites (the only exception were two specimens that lacked a gap in posterior row of tergites 4 and 5). Numbers of trichomes, including the ornamental trichomes *a*, varied also with no distinct geographic pattern discernible. The wide distribution, and presence of the species on islands on both sides of the continent points to the possible movement of the species by birds and or wind.

In the course of this study, collections from the Hamersley Ranges of Western Australia previously identified as *Unixenus mjoebergi* were found to have a number of important differences requiring erection of a new species, *Unixenus karajinensis* sp. n.

#### Unixenus karajinensis sp. n.

urn:lsid:zoobank.org:act:8D5DDD19-2571-437F-AE9D-74B932AD9ABB

http://species-id.net/wiki/Unixenus_karajinensis

Figs 1C 4 5 

##### Holotype.

Male, Wittenoom Gorge, asbestos mine, WA, 22°19'S, 118°19'E, 20 May 1977, WD Temperton, WAM T71106, mounted on slide.

##### Paratypes.

Six females same data as holotype, on six slides, WAM T116452–7; four females and three males from Tom Price, WA, 22°41'S, 117°47'E, 727 m, 6 February 1978, CE Chobanoff, females: WAM T116458–61, males: WAM T116462–64, mounted on slides.

##### Other material.

Additional specimens, same data as holotype, WAM T116451; additional specimens, same data as paratypes from Tom Price, WAM T116465; AM collection from Hamersley Ranges, WA, 10 km north from Tom Price turnoff along Nanutarra - Wittenoom Rd, on left side, 22°32'21"S, 117°38'01"E, 25 May–4 June 2004, M. Bulbert et al., pitfall traps, AM KS111219.

##### Diagnosis.

Differs from *Unixenus mjoebergi* in longer and thinner tergal trichomes, 6 pairs of coxal glands in males on leg pairs 6–11, telotarsus with anterior spinous projection shorter than claw, 8 ornamental trichomes *c* each side. Antennal articles VI and VII with distinctive notched appearance at distal edge, arrangement of sensilla in article VI with setiform sensillum anterior to 3 basiconic sensilla. Number of setae on coxae 3–13 varies more widely from 1–6 in contrast to 2–3 in *Unixenus mjoebergi*. The hooked caudal trichomes have double barbs proximal to the hooks, last sternal plate with 2 setae.

**Figure 4.**
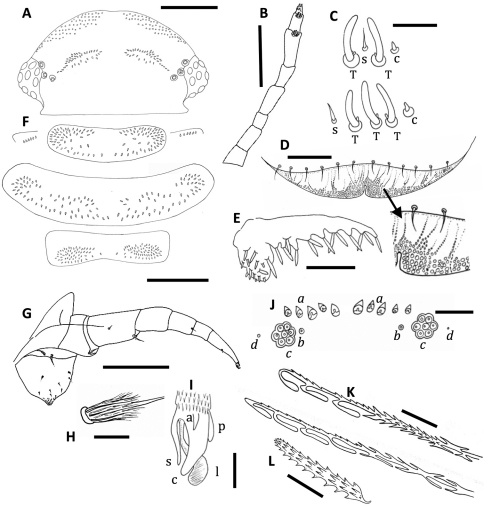
*Unixenus karajinensis* sp. n., holotype, WAM T71106 **A** Head, dorsal view showing arrangement of ocelli, position of trichobothria and trichome insertions **B** Right antenna **C** Details of sensilla on antennal articles VI and VII, sensilla type indicated as follows: coeloconic (c), setiform (s), thick basiconic (T), article VI sensilla lower row **D** Clypeo-labrum with enlargement to show papillae **E** Right palp of gnathochilarium showing long lateral palp, medial palp and simple sensilla **F** Collum, tergite 2 and tergite 10 showing pattern of trichome insertions **G** Left leg 2 showing chaetotaxy on leg segments and penis **H** Details of seta on coxa, prefemur and femur **I** Anterior view of left telotarsus showing anterior spinous projection (s), claw (c) with anterior (a) and posterior (p) processes and lamella (l) **J** Pattern of ornamental trichomes *a, b, c* and circular indentations *d* **K** Distal portion of typical hooked trichomes showing double headed barbs **L** Trichome from tergite 2. Scale bars: **A, B** & **F** = 200 µm; **G** = 100 µm; **E** = 50 µm; **D** = 40 µm; **C, J** & **K** = 20 µm; **H** & **I** = 10µm.

**Figure 5.**
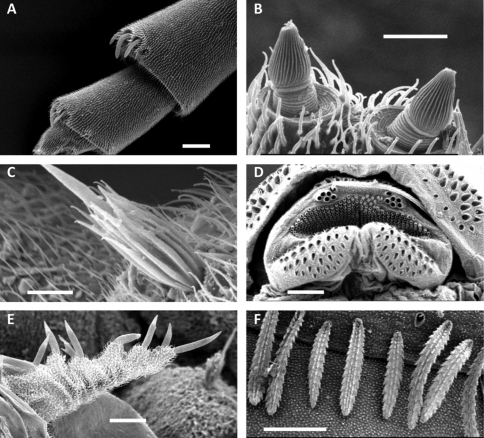
*Unixenus karajinensis* sp. n., Wittenoom **A** Antennal articles VI and VII showing notched distal edge **B** Detail of sensory cones on tip antennal article VIII **C** Ridged funicle of seta of coxa, prefemur and femur **D** Posterior view of stadium VII subadult, showing caudal trichome and associated trichome insertions **E** Lateral palp of gnathochilarium showing simple sensilla **F** Detail of tergal barbed trichomes. Scale bars: **A** = 20 µm, **B** & **C** = 5 µm, **D** & **F** = 50 µm, **E** = 20 µm.

##### Etymology.

The Australian aboriginal Banyjima people’s name for the Hamersley Ranges is Karajini.

##### Description.

Length of both sexes 2.8–3.3 mm, caudal bundle 0.3 mm.

Colouration in alcohol both sexes yellow brown with trichomes including caudal bundle medium to dark brown, long ornamental trichomes darker.

Head with 8 ocelli each side: 4 dorsal, 4 lateral (1 anterior, 2 medial and 1 posterior). Vertex with 2 posterior groups of trichomes arranged in 3 oblique rows (Fig. 4A). Number of trichomes varies, holotype with 40+38 trichomes, comprising: 8+8 (anterior rows), 21+20 (middle rows) and 11+10 (posterior rows). Total numbers each side varied: Tom Price paratypes 27–45 (n = 7); Wittenoom paratypes 25–35 (n = 6). Overall, both sexes showed highly variable number vertex trichomes (51–85), with asymmetrical pattern of 1–2 trichomes difference (occasionally up to 5). Trichobothria equal in size, arranged in shape of isosceles triangle with greater width *a*–*c*.

Antennae with proportions of 8 articles and 4 sensory cones typical of other species in genus (Figs 4B and 5B). Antennal articles VI and VII with distinctive notched appearance at distal edge (Fig. 5A), article VI with 3 thick basiconic sensilla of equal length, coeloconic sensillum posterior to basiconic sensilla, setiform sensillum anterior to basiconic sensilla. Antennal segment VII typical of genus with 1 coeloconic sensillum to the posterior followed anteriorly by 2 thick basiconic sensilla of similar height; 1 setiform sensillum between the basiconic sensilla (Fig. 4C). Clypeo-labrum with 12 setae along posterior margin, anterior margin with no dentition lateral to median cleft, two thirds surface covered with spherical papillae, papillae reducing in size to the posterior margin and lacking hairs (Fig 4D). Gnathochilarium with length lateral palp at least 2 X diameter medial palp; lateral palp with 13 cylindrical sensilla, medial palp 21 sensilla (Figs 4E and 5E).

Collum with almost symmetrical arrangement of trichomes, posterior row of trichomes with broad medial gap, extending to lateral clusters of trichomes, anteriorly a narrowing band of trichomes extending from each lateral cluster towards midline, scattered trichomes between posterior row and anterior bands (Fig. 4F). Number of trichomes 56L + 59R in holotype male; variation common with range per side: Wittenoom female paratypes 31–46 (n = 6); Tom Price paratype females 33–47 (n = 4), paratype males 37–43 (n = 3). Small lateral protuberances each with row of 6 forward-facing trichomes in holotype, varying between 4–6 in Tom Price and Wittenoom paratypes.

Trichomes of tergites 2–9 arranged on posterior half of tergite, 1 distinct posterior row with medial gap, merging with clusters laterally. Further trichomes anteriorly, loosely arranged in 2 to 3 rows. Anterior trichomes directed towards head while remaining trichomes directed posteriorly. Number of trichomes on tergite 2 in holotype male 61L+54R, variation common with range per side: Wittenoom paratype females 36–49 (n = 4); Tom Price paratype females 38–64 (n = 3), paratype males 49–52 (n = 2). Tergite 10 with wide medial gap, large clusters of trichomes either side, posterior rows forming part of each cluster (Fig 4F). Conical pleural projections along each side associated with tergites 2–10, each with dense cluster of trichomes. Tergal and pleural trichomes, long and thin with 2 internal rows projections (Figs 4L and 5F), tergal trichomes increasing in length posteriorly.

Legs 1 and 2 without trochanter, leg 1 also lacks tarsus 1. Trochanter and postfemur and tarsus 1 lack setae. Chaetotaxy as follows: coxa 1, 1 seta, coxa 2, 2 setae, coxae 3–13, 1-6 setae; prefemur, tibia and tarsus 2 with 1 seta, femur with 2 setae (Fig. 4G). Last sternal plate with 2 setae. Coxa, prefemur and femur with bi-articulate setae similar to those for *Unixenus mjoebergi* with longitudinal ridges on basal funicle, each ridge extending distally in a long, thin projections which surround the base of the flagellum (Figs 4H and 5C), setae of tibia and tarsus 2 setiform. Telotarsus bearing anterior spinous projection shorter than claw which bears posterior and anterior processes, large lamella process present (Fig. 4I). Males with 6 pairs coxal glands, leg pairs 6–11.

Telson with ornamental trichomes arranged almost symmetrically with 5 trichomes (some variants with 4–8) *a*, 1*b*, and 8*c* each side of midline (Fig. 4J). Insertion points vary in size with *a* and *c* bigger than *b*. Single caudal bundle of hooked trichomes with 2–4 hooks and barbed stems. Double barbs of stem showing both distal- and proximal-facing barbs (Fig. 4K). Double barbs start immediately below hooks on 4 hook trichomes, with simple distal-facing barbs before first double barb on 2–3 hook trichomes. In immature stadia, two clusters of short barbate trichomes with same structure as ornamental trichomes *a* found ventral to caudal bundle (Fig. 5D). These clusters also observed in other species in the family Polyxenidae. They displace laterally after moulting to become pleural projections.

##### Distribution.

This species is only known from three sites in the Hamersley Ranges, WA (Fig. 8). Both males and females were collected at each location. Since collection, the asbestos mine at Wittenoom has been closed and access restricted to the area that includes the type locality.

##### Remarks.

The widespread distributions of *Unixenus attemsi*, *Unixenus mjoebergi* and *Unixenus corticolus* sp. n. are not unexpected as their small size, bristles and very light weight make it probable that they are blown by the wind or become attached to bird feathers. Unexpectedly *Unixenus karajinensis* sp. n. appears to be limited to a single mountain range, although further sampling may extend the distribution.

#### Unixenus corticolus sp. n.

urn:lsid:zoobank.org:act:9AD17CAD-DE6A-4E67-B0B4-F418D0A889D0

http://species-id.net/wiki/Unixenus_corticolus

Figs 1D 6 7 

##### Holotype.

Male, Deep Lead Flora and Fauna Park, near Stawell, Vic, 37°00'38"S, 142°44'19"E, 17 June 2005, M. Short and C. Huynh, in *Eucalyptus* bark, mounted on slide, MV K-11507.

##### Paratypes.

One male, five females, same data as holotype, mounted on slides, male: MV K-11508, females 1–5: MV K-11509–13; one male and one female from Tidal River, Wilson's Promontory, Vic, 39°01'54"S, 146°18'49"E, 1 November 2005, C. Huynh, in *Melaleuca* bark, mounted on slides, male: MV K-11514, female: MV K-11515.

##### Other material examined.

All collected by M. Short and C. Huynh: Ararat Hills Park, Vic, 37°14'30"S, 142°54'30"E, 17 June 2006, in *Eucalyptus* bark; Flinders, Vic, 38°28'54"S, 145°01'22"E, 25 August 2011, in *Melaleuca* bark; Pt. Addis, Vic, 38°22'27"S, 144°14'42"E, 21 August 2011, in *Eucalyptus* bark; Tathra, NSW, 36°43'35"S, 149°59'8"E, 30 December 2007, in *Melaleuca* bark; Lakes Entrance, Vic, 37°52'45"S, 146°10'49"E, 28 December 2007, in *Melaleuca* bark; Sunnyside Beach, Mornington, Vic, 38°12'06"S, 145°03'41"E, 17 May 2008, in *Melaleuca* bark; Launceston, Tas, 41°27'S, 147°09'E, 1 February 2008, in private garden, bark mulch; Holey Plains State Park, Vic, 38°13'S, 146°53'E, 20 April 2008, exuviae, in *Eucalyptus* bark; Narawntapu National Park, Tas, 42°55'07"S, 147°49'23"E, 31 January 2008, in *Eucalyptus* bark; Cataract Gorge, Launceston, Tas, 41°27'S, 147°09'E, 1 February 2008, in *Eucalyptus* bark.

##### Etymology.

Adjective; this species is almost always found under bark, rarely in litter, in contrast to other species in the genus.

##### Diagnosis.

4 basiconic sensilla on antennal article VI, basiconic sensillum 4 posterior to coeloconic sensillum, short lateral palps on gnathochilarium (1.5 X diameter of medial palp), 1 seta on femur and no setae on tibia, 5 ornamental trichomes *c* each side, caudal hooked trichomes with 3–6 hooks. Setae of coxa, prefemur and femur similar to that of *Unixenus mjoebergi* but with slightly curving and fewer ridges on funicle.

##### Description.

Length of both sexes 2.4–3.4 mm, caudal bundle 0.45 mm.

Colourdark grey with broad unpigmented band dorsally along midline. Trichomes unpigmented, appearing translucent white in live specimen, long ornamental trichomes *c* darkly pigmented in contrast to trichomes of caudal bundle that appear white in live specimens.

**Figure 6.**
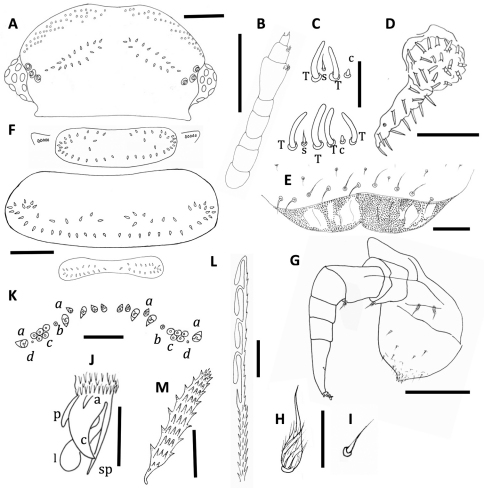
*Unixenus corticolus* sp. n., holotype, MV K-11507 **A** Head, dorsal view showing arrangement of ocelli, position of trichobothria and trichome insertions **B** Right antenna **C** Details of sensilla on antennal articles VI and VII, sensilla type indicated as follows: coeloconic (c), setiform (s), thick basiconic (T), article VI sensilla lower row **D** Left palp of gnathochilarium showing long lateral palp, medial palp and simple sensilla **E** Clypeo-labrum **F** Collum, tergite 2 and tergite 10 showing pattern of trichome insertions **G** Leg 2 showing chaetotaxy and penis **H** Details of seta of coxa, prefemur and femur **I** Setiform sensillum, tarsus 2 **J** Anterior view of telotarsus showing anterior spinous projection (s), claw (c) with anterior (a) and posterior (p) processes and lamella (l) **K** Pattern of ornamental trichomes *a, b, c* and circular indentations *d* **L** Hooked caudal trichome **M** Tergal trichome. Scale bars: **B** & **G** = 150 µm; **A** & **F** = 100 µm, **D** = 50 µm, **E** & **K** = 40 µm; **M** = 30 µm; **C**, **H** & **I** (shared bar), **J** & **L** = 10 µm.

Head (Fig. 6A) with 8 ocelli each side: 4 dorsal, 4 lateral (1 anterior, 2 medial and 1 posterior). Vertex with 2 posterior groups of trichomes arranged in 2 oblique rows, separated medially by a broad space. Number of trichomes varies, holotype with 10L+10R (anterior rows) and 7L+6R (posterior rows), total each side 17L and 16R. Number of trichomes per side varies 14–18, with no differences between sexes, asymmetrical pattern common with maximum difference 2 trichomes. Trichobothria equal in size, arranged in triangle with angle at b >120^o^, distance a–b slightly shorter than distance b–c.

**Figure 7.**
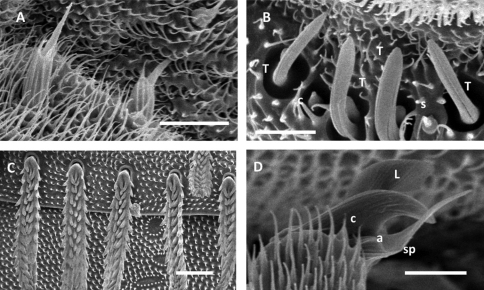
*Unixenus corticolus* sp. n., adult male, Deep Lead **A** Setae on coxa **B** Sensilla on antennal article VI, thick basiconic (T), coeloconic (c), setiform (s) **C** Detail of barbed tergal trichomes **D** Detail of telotarsus showing anterior spinous projection (sp), claw (c), lamella process (l), anterior process (a). Scale bars: **A** = 10 µm, **B** & **D** = 5 µm, **C** = 20 µm.

Antennae with proportions of 8 articles and 4 sensory cones typical of other species in genus *Unixenus* (Fig. 6B). Antennal article VI with 4 thick basiconic sensilla of equal length, coeloconic sensillum between basiconic sensilla 3 and 4, setiform sensillum between basiconic sensilla 1 and 2. Antennal segment VII typical of genus with 1 coeloconic sensillum to the posterior followed anteriorly by 2 thick basiconic sensilla of similar height; 1 setiform sensillum between the basiconic sensilla (Figs 6C and 7B). Clypeo-labrum covered in small spherical papillae typical of genus *Unixenus*, anterior edge with median cleft, no lamella teeth, posterior margin 8–10 setae (Fig. 6E). Gnathochilarium with length lateral palp 1.5 X diameter of medial palp. Lateral palp with 13 cylindrical sensilla, medial palp 21 sensilla (Fig. 6D).

Collum with almost symmetrical arrangement of trichomes, 2 main rows of trichomes each side of medial gap with small number of trichomes between rows, rows linked laterally with small cluster of trichomes (Fig. 6F). Trichomes 24L+23R in holotype, varying between 23–28 per side in paratypes. Small lateral protuberances each with row of 5 forward facing trichomes in holotype, 3–7 in paratypes. In tergites 2–10, trichomes arranged on posterior half of tergite in 2 loose rows with small clusters laterally (Fig. 6F). Trichomes in anterior row directed towards head, those of posterior row directed posteriorly. Trichome number on tergite 2 variable: 26L+26R in holotype, 24–34 each side in paratypes. Conical pleural projections along each side associated with tergites 2–10. Tergal trichomes, all short, barbate and thicker than those of *Unixenus mjoebergi* and *Unixenus karajinensis* with three internal longitudinal rows of projections (Figs 6M and 7C), pleural trichomes slightly longer.

Legs 1 and 2 without trochanter, leg 1 also lacks tarsus 1. Trochanter, postfemur, tibia and tarsus 1 lack setae. Chaetotaxy as follows: coxa 1, one seta, coxa 2–3, 2–3 setae, coxa 3–10, 2–4 setae, coxa 13, 0–2 setae (males 2 setae, females 0 setae); prefemur, femur, and tarsus 2 with single seta (Fig. 6G). Structure of setae of coxa, prefemur, femur similar to that of *Unixenus mjoebergi* and *Unixenus karajinensis* but with less ridging on funicle and fewer projections, ridging slightly curved around funicle (Figs 6H and 7A); seta of tarsus 2 setiform (Fig. 6I). Telotarsus bearing anterior very thin spinous projection longer than claw. Claw bears anterior and posterior slender processes, large lamella process (Figs 6J and 7D). Males with 2 pairs coxal glands on leg pairs 8–9.

Ornamental trichomes of telson with 4–6 trichomes *a*, 1*b*, and 5*c* (comprising 2 long, 1 medium and 2 shorter dark brown barbate trichomes) each side of midline (Fig. 6K). Caudal bundle trichomes with 3–6 hooks, distal-facing barbs along the stem (Fig. 6L).

##### Distribution.

**Figure 8.**
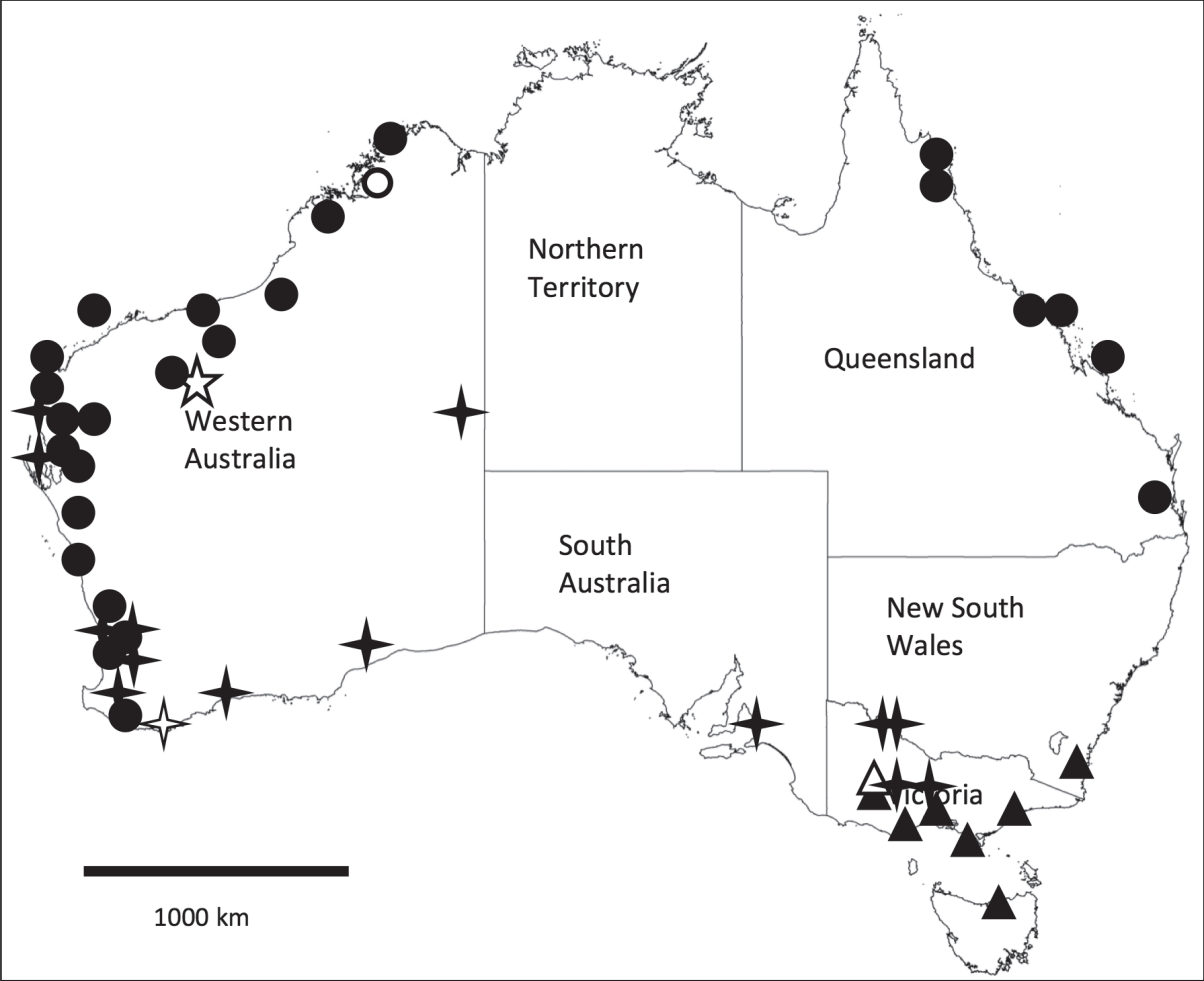
Map of Australia with states indicated, showing distribution of four *Unixenus* species. *Unixenus mjoebergi* (Verhoeff, 1924): filled circles, type region shown as open circle; *Unixenus attemsi* Nguyen Duy-Jacquemin and Conde, 1967: filled 4 point star, type locality shown as open 4 point star; *Unixenus karajinensis* sp. n.: type locality shown as open 5 point star; *Unixenus corticolus* sp. n.: filled triangles, type locality shown as open triangle.

Specimens of this species have been found at a number of locations some distance from each other in southern Australia (Fig. 8). Specimens were collected from under bark of *Eucalyptus*, *Melaleuca* and *Leptospermum*. The species was found once only on the ground, in litter formed from mulched bark. Males and females were collected at each location. No specimens were found in museum collections.

##### Remarks.

Although this species is similar in many ways to *Unixenus mjoebergi*, it appears to have different habitat requirements as it is rarely found in litter, and then only in bark mulch. This would explain the absence of the species from museum collections that are mainly the result of pitfall trapping or extraction from litter.

### Key to described species of *Unixenus*

The type species from India, *Unixenus padmanabhii*, is not included as insufficient details are known:

**Table:** 

1a.	Presence of unridged setae on legs	2
1b.	Presence of leg setae with ridges and spiny projections	3
2a.	One small seta on tibia of at least legs 1–5	*Unixenus broelemanni*
2b.	No setae on tibia	4
3a.	Presence of 3 ornamental trichomes *c* per side	*Unixenus mjoebergi*
3b.	Presence of more than 3 ornamental trichomes *c* per side	5
4a.	Telotarsus with more than 2 processes on claw	*Unixenus attemsi*
4b	Telotarsus with 1 process only on claw	*Unixenus vuillaumei*
5a.	5 ornamental trichomes *c* per side, 2 pairs coxal glands in male	*Unixenus corticolus* sp. n.
5b.	8 ornamental trichomes *c* per side, 6 pairs coxal glands in male	*Unixenus karajinensis* sp. n.

## Supplementary Material

XML Treatment for Unixenus

XML Treatment for Unixenus attemsi

XML Treatment for Unixenus mjoebergi

XML Treatment for Unixenus karajinensis

XML Treatment for Unixenus corticolus
